# Development and validation of a preoperative difficulty scoring system for endoscopic resection of gastric gastrointestinal stromal tumor: a multi-center study

**DOI:** 10.1007/s00464-023-10106-w

**Published:** 2023-05-16

**Authors:** Luojie Liu, Mei Han, Dongtao Shi, Qinghua Wang, Yunfu Feng, Fenying Lu, Rui Li, Xiaodan Xu

**Affiliations:** 1 Department of Gastroenterology, Changshu Hospital Affiliated to Soochow University, Suzhou, China; 2 Department of Gastroenterology, The First Affiliated Hospital of Soochow University, Suzhou, China; 3 Department of Health Management Center, The First Affiliated Hospital of Soochow University, Suzhou, China; 4 Department of Gastroenterology, No. 1 People’s Hospital of Kunshan, Suzhou, China; 5 Department of Gastroenterology, No. 2 People’s Hospital of Changshu, Suzhou, China

**Keywords:** Difficulty scoring system, Endoscopic resection, Gastrointestinal stromal tumors

## Abstract

**Background:**

Endoscopic resection (ER) is a promising technique for resecting gastric gastrointestinal stromal tumors (gGISTs); however, ER is technically challenging. This study aimed to develop and validate a difficulty scoring system (DSS) to determine the difficulty for ER of a gGIST.

**Methods:**

This retrospective study enrolled 555 patients with gGISTs in multi-centers from December 2010 to December 2022. Data on patients, lesions, and outcomes of ER were collected and analyzed. A *difficult case* was defined as an operative time ≥ 90 min, or the occurrence of severe intraoperative bleeding, or conversion to laparoscopic resection. The DSS was developed in the training cohort (TC) and validated in the internal validation cohort (IVC) and external validation cohort (EVC).

**Results:**

The difficulty occurred in 97 cases (17.5%). The DSS comprised the following: tumor size ≥ 3.0 cm (3 points) or 2.0–3.0 cm (1 point); location in the upper third of the stomach (2 points); invasion depth beyond the muscularis propria (2 points); lack of experience (1 point). The area under the curve (AUC) of DSS in IVC and EVC was 0.838 and 0.864, respectively, and the negative predictive value (NPV) was 0.923 and 0.972, respectively. The proportions of difficult operation in easy (score 0–3), intermediate (score 4–5), and difficult (score 6–8) categories were 6.5%, 29.4%, and 88.2% in the TC, 7.7%, 45.8%, and 85.7% in the IVC, and 7.0%, 29.4%, and 85.7% in the EVC, respectively.

**Conclusions:**

We developed and validated a preoperative DSS for ER of gGISTs based on tumor size, location, invasion depth, and endoscopists’ experience. This DSS can be used to grade the technical difficulty before surgery.

**Supplementary Information:**

The online version contains supplementary material available at 10.1007/s00464-023-10106-w.

Gastric gastrointestinal stromal tumors (gGISTs) are the most common type of submucosal tumors (SMTs) found in the stomach. Standard preoperative diagnosis methods for gGIST include white light endoscopy, endoscopic ultrasound (EUS), and CT examination [1, 2]. According to the National Comprehensive Cancer Network (NCCN) guidelines, regular endoscopic follow-up is recommended for small gGISTs without a high risk of malignant changes under EUS [3]. However, due to the malignant behavior of gGISTs, European and Japanese guidelines recommend resection for all pathologically confirmed cases [4, 5].

Advancements in endoscopic resection (ER) techniques offered a promising opportunity for treating low-risk gGISTs, with the advantages of faster postoperative recovery, shorter hospital stays, and lower cost [6–8]. Nevertheless, ER of gGISTs can be complex and carries the risk of severe intraoperative bleeding, tumor rupture, and conversion to laparoscopic resection. To improve the quality of gGISTs ER outcomes, evaluating the level of operative difficulty before the surgery is essential.

The difficulty scoring system (DSS) is an effective tool for objectively evaluating the degree of surgical difficulty, which has been widely used for laparoscopic liver resection, splenectomy, and resection of digestive tract malignant tumor [9–11]. Therefore, this study aimed to develop a DSS that can objectively evaluate the difficulty of ER of gGISTs and to assess its ability of this model to predict the difficulty of this procedure.

## Material and methods

### Patients

We retrospectively analyzed the data of patients who underwent ER for gGISTs at the First Affiliated Hospital of Soochow University from December 2010 to December 2022 and randomly assigned all patients to the training cohort (TC) and internal validation cohort (IVC) on a 7:3 basis. For external verification, we collected data of patients with gGISTs who received endoscopic therapy at Changshu Hospital Affiliated to Soochow University, No.1 People’s Hospital of Kunshan, and No.2 People’s Hospital of Changshu from January 2017 to December 2022. The principal inclusion criteria were: (1) postoperative pathological and immunohistochemical diagnosis as a gGIST; (2) preoperative blood routine, hemagglutination, and electrocardiogram tests were normal; (3) patients had no lymph nodes or distant metastasis. The exclusion criteria were: (1) lesions with a high risk of malignant changes under EUS; (2) patients with synchronous lesions at different sites; (3) patients with multiple lesions in the stomach; (4) patients who cannot tolerate anesthesia and surgery due to poor cardiopulmonary function; (5) the patient’s medical record was incomplete. The ethics committee approved the study protocol for clinical research at our institutes. All patients were informed about the risks and benefits of ER and signed a written informed consent for the procedure.

### Endoscopic equipment and procedures

We used three ER techniques according to the lesion: endoscopic submucosal dissection (ESD), endoscopic full-thickness resection (EFTR), and submucosal tunnel endoscopic resection (STER). Details of the ER procedures were described previously [12–14]. Procedures were fulfilled by endoscopists with a different experience in ER of gGISTs; however, all cases were performed by senior endoscopists, each of whom had performed more than 5,000 gastroscopy and colonoscopy procedures and more than 200 EMR procedures for gastrointestinal polyps before undergoing ER of gGISTs. When an endoscopist had performed more than 50 gGISTs ER, he or she was defined as an experiened endoscopist in our study. All patients received general anesthesia and endotracheal intubation. ER was performed by a dual knife (KD-650L; Olympus, Japan), an insulated-tip knife (KD-611L; Olympus, Japan), or a combination. A single-channel endoscope (GIF-Q260J, Olympus, Japan) with a transparent cap attached to the endoscope tip was used. A High-frequency electric coagulation and electrocautery device (ERBE VIO 200D) was used for energy output. Other equipment included: metallic clips, nylon loops (LeClampTM Loop-20 and Loop-30; Leo, Changzhou, China), over-the-scope clips(OTSC), injection needles, hot biopsy forceps, and carbon dioxide insufflator.

### Postoperative management

Postoperative specimens were fixed in 10% formalin solution, and immunohistochemical staining (CD117, CD34, Dog-1, etc.) was performed to confirm the diagnosis. Usually, all patients underwent nasogastric decompression after surgery and were required to fast for two days (3 days or longer for EFTR patients, according to the postoperative condition). Blood routine, CRP, and/or calcitonin examination were performed after surgery, and proton pump inhibitors, gastric mucosal protective agents, fluid replacement, and nutritional support were given to all patients. When patients have abdominal pain or muscle tension, a CT or orthostatic X-ray examination is performed to rule out a postoperative perforation and undergo antibiotic therapy or surgical treatment depending on the condition. Antibiotics were recommended for patients with either intraoperative perforation or postoperative infection.

### Data collection

Each patient’s information, including age, gender, primary symptom, history of smoking or drinking, past medical history, body mass index (BMI), American Society of Anaesthesiologists (ASA) score [15], tumor size, location, shape, invasion depth, boundary, procedure duration, intraoperative and postoperative complications, type of ER technique, R0 resection rates, modified National Institutes of Health (NIH) risk criteria [16], postoperative hospitalization, and postoperative fasting days, were obtained from electronic medical records of our institutes.

### Definitions

A difficult case was defined as an operative time ≥ 90 min, or the occurrence of severe intraoperative bleeding, or conversion to laparoscopic resection. The operative time was defined from the start of the submucosal injection to the completion of the closure of the defect. Severe intraoperative bleeding was defined as repeated endoscopic hemostasis with a postoperative hemoglobin drop of > 2 g/dL or a requirement for surgical assistance [17, 18]. Characteristics of tumors were judged according to the preoperative endoscopic ultrasound examination or abdominal enhanced CT. Postoperative complications mainly include delayed bleeding, delayed perforation, and postoperative infection. Delayed bleeding was defined as clinical evidence of bleeding after ER, characterized by hematemesis or melena, a decrease in hemoglobin levels greater than 2.0 g/dl within 24 h, or requiring endoscopic therapy [19]. Delayed perforation was confirmed by X-ray or computed tomography. Postoperative infection was defined as postoperative body temperature exceeding 37.5℃ and/or accompanied by increased inflammatory indicators such as blood routine, CRP, or calcitonin [20].

### Statistical analysis

Categorical variables were expressed as frequencies and percentages, and the Chi-square test or Fisher exact test was used for comparison between groups. Continuous variables were expressed as median and interquartile ranges (IQR), and the Mann–Whitney U test was performed for comparison between the two groups. The Bonferroni correction method was used for the pairwise comparison of data between multiple groups. A preliminary univariate analysis was performed to select risk factors for the difficult procedure. Predictors with a P-value < 0.05 in the univariate analysis were subjected to a multivariate analysis using the logistic regression model. A P-value < 0.05 was considered statistically significant. DSS was developed based on the β value of the regression coefficient in the multivariate analysis. We defined the variable with the smallest regression coefficient as 1 point and divided the other variables by the multiple of the smallest variable to define the corresponding score. The discrimination of the DSS was assessed by calculating the area under the curve (AUC) of the receiver operating characteristic (ROC) with a 95% confidence interval (CI). We assessed the calibration of the DSS with the Hosmer–Lemeshow goodness-of-fit test. From the continuous score in the TC, we classified patients into three categories according to the level of difficulty (easy, intermediate, and difficult). The rate of difficult operations for ER of gGISTs in each group was compared in TC, IVC, and EVC. Statistical analyses were conducted using SPSS version 26 (Chicago, IL, USA).

## Results

### General characteristics of patients and lesions

**Fig. 1:**
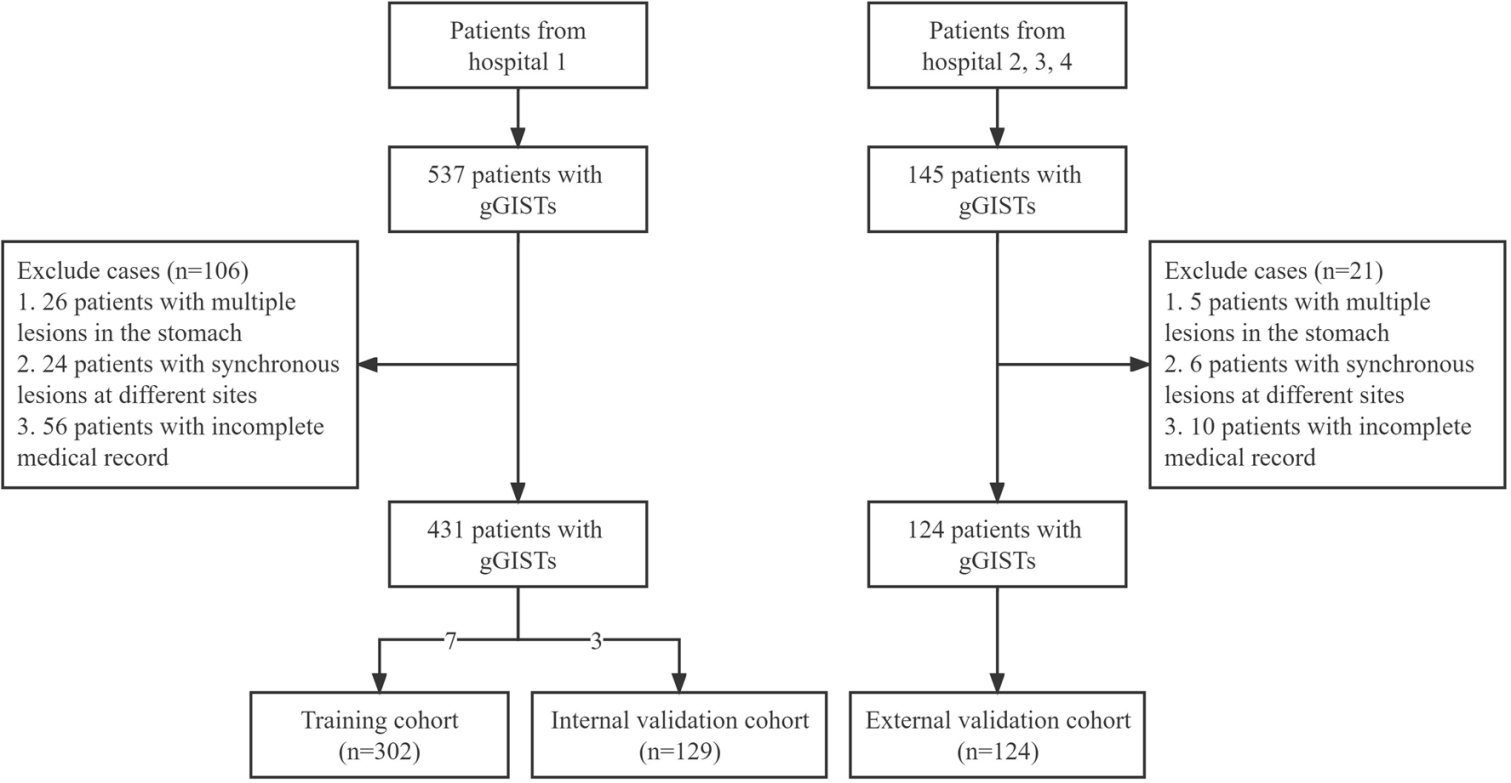
Flow chart of the study. gGISTs: gastric gastrointestinal stromal tumors

A flow chart of the study protocol is shown in Fig. 1. The characteristics of 431 gGISTs in the TC and the IVC are shown in Table 1. The difference in patient and lesion characteristics were not significant between the TC and the IVC (*P* > 0.05). The data of the EVC and its comparison with the TC are shown in Supplementary Table 1. There were statistically significant differences between the two cohorts in terms of gender, tumor location, tumor boundary, and tumor size (*P* < 0.05), while no significant statistical difference were observed between the two groups in other aspects (*P* > 0.05).

**Table 1:** Baseline characteristics of the patients and lesions

Variable	All (N = 431)	Training cohort (N = 302)	Internal validation cohort (N = 129)	*P*-value
Gender, n (%)				0.077
Male	195 (45.2)	145 (48.0)	50 (38.8)	
Female	236 (54.8)	157 (52.0)	79 (61.2)	
Age, yesrs, n (%)				0.360
< 60	216 (50.1)	147 (48.7)	69 (53.5)	
≥ 60	215 (49.9)	155 (51.3)	60 (46.5)	
Primary symptom, n (%)				0.675
Asymptomatic	89 (20.6)	65 (21.5)	24 (18.6)	
Abdominal discomfort	333 (77.3)	230 (76.2)	103 (79.8)	
Hemorrhage	9 (2.1)	7 (2.3)	2 (1.6)	
Smoking, n (%)				0.911
Yes	132 (30.6)	92 (30.5)	40 (31.0)	
No	299 (69.4)	210 (69.5)	89 (69.0)	
History of drinking, n (%)				0.404
Yes	91 (21.1)	67 (22.2)	24 (18.6)	
No	340 (78.9)	235 (77.8)	105 (81.4)	
Hypertension, n (%)				0.769
Yes	138 (32.0)	98 (32.5)	40 (31.0)	
No	293 (68.0)	204 (67.5)	89 (69.0)	
Coronary disease, n (%)				0.775
Yes	80 (18.6)	55 (18.2)	25 (19.4)	
No	351 (81.4)	247 (81.8)	104 (80.6)	
Diabetes, n (%)				0.616
Yes	110 (25.5)	75 (24.8)	35 (27.1)	
No	321 (74.5)	227 (75.2)	94 (72.9)	
ASA score, n (%)				0.209
I	356 (82.6)	253 (83.8)	103 (79.8)	
II	74 (17.2)	49 (16.2)	25 (19.4)	
III	1 (0.2)	0	1 (0.8)	
BMI, kg/m^2^, n (%)				0.903
< 18.5	76 (17.6)	53 (17.5)	23 (17.8)	
18.5–23.9	208 (48.3)	144 (47.7)	64 (49.6)	
≥ 24.0	147 (34.1)	105 (34.8)	42 (32.6)	
Location, n (%)				0.559
Upper	306 (71.0)	210 (69.5)	96 (74.4)	
Middle	84 (19.5)	61 (20.2)	23 (17.8)	
Lower	41 (9.5)	31 (10.3)	10 (7.8)	
Location, n (%)				0.852
Lesser curvature	148 (34.3)	107 (35.4)	41 (31.8)	
Greater curvature	35 (8.1)	25 (8.3)	10 (7.8)	
Anterior	171 (39.7)	116 (38.4)	55 (42.6)	
Posterior	77 (17.9)	54 (17.9)	23 (17.8)	
Shape, n (%)				0.121
Regular	383 (88.9)	273 (90.4)	110 (85.3)	
Irregular	48 (11.1)	29 (9.6)	19 (14.7)	
Invasion depth, n (%)				0.386
MM	95 (22.0)	70 (23.2)	25 (19.4)	
MP	270 (62.6)	190 (62.9)	80 (62.0)	
MP-ex	66 (15.3)	42 (13.9)	24 (18.6)	
Boundary, n (%)				0.267
Clear	379 (87.9)	269 (89.1)	110 (85.3)	
Unclear	52 (12.1)	33 (10.9)	19 (14.7)	
Size, cm, n (%)				0.418
≥ 3.0	65 (15.1)	42 (13.9)	23 (17.8)	
2.0–3.0	115 (26.7)	85 (28.1)	30 (23.3)	
< 2.0	251 (58.2)	175 (57.9)	76 (58.9)	

*ASA* American Society of Anesthesiologists; *BMI* body mass index; *MM* muscularis mucosae; *MP* muscularis propria; *MP-ex* MP with exophytic growth

### Procedural outcomes related to ER of gGISTs

There were 49 (16.2%) and 30 (23.3%) difficult cases in the TC and IVC (Table 2). Of the 431 patients, 288 (66.8%) were performed by experienced endoscopists, and 143 (33.2%) were performed by less-experienced endoscopists. Patients treated with ESD, EFTR, and STER accounted for 49.2%, 49.2%, and 1.6%, respectively. The median operative time was 59.0 min (IQR, 45.0–78.0 min). The conversion rate was 3.2% (14/431), the severe intraoperative bleeding rate was 3.2% (14/431), the R0 resection rate was 91.4% (394/431), and the postoperative complications rate was 13.5% (58/431). The median postoperative fasting days and postoperative hospitalization days were 3.0 days and 6.0 days. There were no differences in ER procedure outcomes between the TC and the IVC (*P* > 0.05) (Table 3). For the 124 cases in the EVC, difficult cases were experienced in 18 (14.5%) cases (Supplementary Table 2), the median operative time was 68.0 min (IQR, 50.0–75.0 min), and the severe intraoperative bleeding rate was 3.2% (4/124). Supplementary Table 3 presents details of the procedural outcomes related to ER of gGISTs in the EVC and compares them with those of the TC. The EVC had longer operative times (median 68.0 vs. 59.0 min, *P* = 0.016) and lower rate of conversion (0% vs. 3.3%, *P* = 0.039) compared to the TC. In other aspects, there was no significant statistical difference observed between the two groups (*P* > 0.05).

**Table 2:** Distribution of cases defined as difficult procedure in training cohorts and internal validation cohort

	Training cohort (N = 302)	Internal validation cohort (N = 129)
Difficult procedure, n (%)	49 (16.2)	30 (23.3)
Long operative time, n (%)	45 (14.9)	28 (21.7)
Severe intraoperative bleeding, n (%)	8 (2.6)	6 (4.7)
Conversion, n (%)	10 (3.3)	4 (3.1)

**Table 3:** Procedural outcomes related to ER of gGISTs

Variable	All (N = 431)	Training cohort (N = 302)	Internal validation cohort (N = 129)	*P*-value
Experience, cases, n (%)				0.166
< 50	143 (33.2)	94 (31.1)	49 (38.0)	
≥ 50	288 (66.8)	208 (68.9)	80 (62.0)	
Endoscopic tecnique, n (%)				0.084
ESD	212 (49.2)	159 (52.6)	53 (41.1)	
EFTR	212 (49.2)	138 (45.7)	74 (57.4)	
STER	7 (1.6)	5 (1.7)	2 (1.6)	
Modified NIH risk criteria, n (%)				0.556
Very low	301 (69.8)	205 (67.9)	96 (74.4)	
Low	88 (20.4)	65 (21.5)	23 (17.8)	
Intermediate	36 (8.4)	27 (8.9)	9 (7.0)	
High	6 (1.4)	5 (1.7)	1 (0.8)	
Operative time, min, median(IQR)	59.0 (45.0, 78.0)	59.0 (45.0, 74.3)	60.0 (25.0, 85.0)	0.297
Conversion, n (%)	14 (3.2)	10 (3.3)	4 (3.1)	1.000
Severe intraoperative bleeding, n (%)	14 (3.2)	8 (2.6)	6 (4.7)	0.283
Postoperative hospitalization, days, median(IQR)	6.0 (5.0, 7.0)	6.0 (5.0, 7.0)	5.0 (4.5, 7.0)	0.333
Postoperative fasting, days, median(IQR)	3.0 (2.0, 3.0)	3.0 (2.0, 3.0)	3.0 (2.0, 4.0)	0.115
R0 resection, n (%)	394 (91.4)	275 (91.1)	119 (92.2)	0.687
Postoperative complications, n (%)	58 (13.5)	46 (15.2)	12 (9.3)	0.099

*ER* endoscopic resection; *gGIST* gastric gastrointestinal stromal tumor; *ESD* endoscopic submucosal dissection; *EFTR* endoscopic full-thickness resection; *STER* submucosal tunnel endoscopic resection; *NIH* National Institute of Health; *IQR* interquartile ranges

### Risk factors for the difficulty of ER of gGISTs

Univariate analysis revealed that tumor location, shape, size, invasion depth, and endoscopists’ experience were risk factors for ER of gGISTs (Table 4). Multivariate analysis showed that the independent predictors for the difficulty of ER of gGISTs in the TC were tumor size, location, invasion depth, and endoscopists’ experience (Table 5). Compared with gGISTs < 2.0 cm, gGISTs of 2.0–3.0 cm significantly increased the ER difficulty (OR 2.344, 95% CI 1.091–6.040, *P* = 0.031), and the strongest risk indicator was gGISTs larger than 3.0 cm (OR 14.271, 95% CI 4.880–41.735, *P* < 0.001). Lesions in the upper third of the stomach exhibited significantly greater difficulty than other sites (OR 5.402, 95% CI 1.054–27.702,* P* = 0.043). A gGIST originating from MP-ex increased the difficulty more than a superficial gGIST (OR 4.433, 95% CI 1.367–14.382, *P* = 0.013). The risk of difficulty for procedures performed by non-experienced endoscopists was significantly higher than that of experienced endoscopists (OR 2.344, 95% CI 1.125–4.884, *P* = 0.023).

**Table 4:** Univariate analysis of the risk factors for the difficulty of ER of gGISTs

Variable	Difficulty (N = 49)	Non-difficulty (N = 253)	*P*-value
Gender, n (%)			0.278
Male	27 (55.1)	118 (46.6)	
Female	22 (44.9)	135 (53.4)	
Age, yesrs, n (%)			0.436
< 60	21 (42.9)	126 (49.8)	
≥ 60	28 (57.1)	127 (50.2)	
Primary symptom, n (%)			0.663
Asymptomatic	10 (20.4)	55 (21.7)	
Abdominal discomfort	37 (75.5)	193 (76.3)	
Hemorrhage	2 (4.1)	5 (2.0)	
Smoking, n (%)			0.716
Yes	16 (32.7)	76 (30.0)	
No	33 (67.3)	177 (70.0)	
History of drinking, n (%)			0.671
Yes	12 (24.5)	55 (21.7)	
No	37 (75.5)	198 (78.3)	
Hypertension, n (%)			0.484
Yes	18 (36.7)	80 (31.6)	
No	31 (63.3)	173 (68.4)	
Coronary disease, n (%)			0.663
Yes	10 (20.4)	45 (17.8)	
No	39 (79.6)	208 (82.2)	
Diabetes, n (%)			0.306
Yes	15 (30.6)	60 (23.7)	
No	34 (69.4)	193 (76.3)	
ASA score, n (%)			0.673
I	40 (81.6)	213 (84.2)	
II	9 (18.4)	40 (15.8)	
III	0	0	
BMI, kg/m^2^, n (%)			0.965
< 18.5	8 (16.3)	45 (17.8)	
18.5–23.9	24 (49.0)	120 (47.4)	
≥ 24.0	17 (34.7)	88 (34.8)	
Location, n (%)			< 0.001
Upper	46 (93.9)	164 (64.8)	
Middle	1 (2.0)	60 (23.7)	
Lower	2 (4.1)	29 (11.5)	
Location, n (%)			0.857
Lesser curvature	20 (40.8)	87 (34.4)	
Greater curvature	4 (8.2)	21 (8.3)	
Anterior	17 (34.7)	99 (39.1)	
Posterior	8 (16.3)	46 (18.2)	
Shape, n (%)			0.023
Regular	40 (81.6)	233 (92.1)	
Irregular	9 (18.4)	20 (7.9)	
Invasion depth, n (%)			0.020
MM	9 (18.3)	61 (24.1)	
MP	27 (55.1)	163 (64.4)	
MP-ex	13 (16.5)	29 (11.5)	
Boundary, n (%)			0.410
Clear	42 (85.7)	227 (89.7)	
Unclear	7 (14.3)	26 (10.3)	
Size, cm, n (%)			< 0.001
≥ 3.0	21 (42.9)	21 (8.3)	
2.0–3.0	15 (30.6)	70 (27.7)	
< 2.0	13 (26.5)	162 (64.0)	
Experience, cases, n (%)			0.001
< 50	26 (53.1)	68 (26.9)	
≥ 50	23 (46.9)	185 (73.1)	

*ER* endoscopic resection; *gGIST* gastric gastrointestinal stromal tumor; *ASA* American Society of Anesthesiologists; *BMI* body mass index; *MM* muscularis mucosae; *MP* muscularis propria; *MP-ex* MP with exophytic growth

**Table 5:** Multivariate analysis of the risk factors for the difficulty of ER of gGISTs and development of DSS

Variable	OR	95%CI	*P*-value	Regression coefficient β	Multiple relationship	Score
Location, n (%)						
Upper	5.402	1.054–27.702	0.043	1.687	1.980	2
Middle	0.407	0.032–5.252	0.491	− 0.899	–	0
Lower	–	–	–	1 (Reference)	–	0
TechnSize, cm, n (%)						
≥ 3.0	14.271	4.880–41.735	< 0.001	2.658	3.120	3
2.0–3.0	2.567	1.091–6.040	0.031	0.943	1.107	1
< 2.0	–	–	–	1 (Reference)	–	0
Experience, cases, n (%)						
< 50	2.344	1.125–4.884	0.023	0.852	1 (Reference)	1
≥ 50	–	–	–	1 (Reference)	–	0
Invasion depth, n (%)						
MP-ex	4.433	1.367–14.382	0.013	1.489	1.748	2
MP	1.297	0.511–3.290	0.584	0.260	–	0
MM	–	–	–	1 (Reference)	–	0
Shape, n (%)						
Irregular	0.964	0.244–3.808	0.959	− 0.036		
Regular	–	–	–	1 (Reference)		

*ER* endoscopic resection; *gGIST* gastric gastrointestinal stromal tumor; *DSS* difficulty scoring system; *MM* muscularis mucosae; *MP* muscularis propria; *MP-ex* MP with exophytic growth

### Development and validation of DSS for ER of gGISTs

**Fig. 2:**
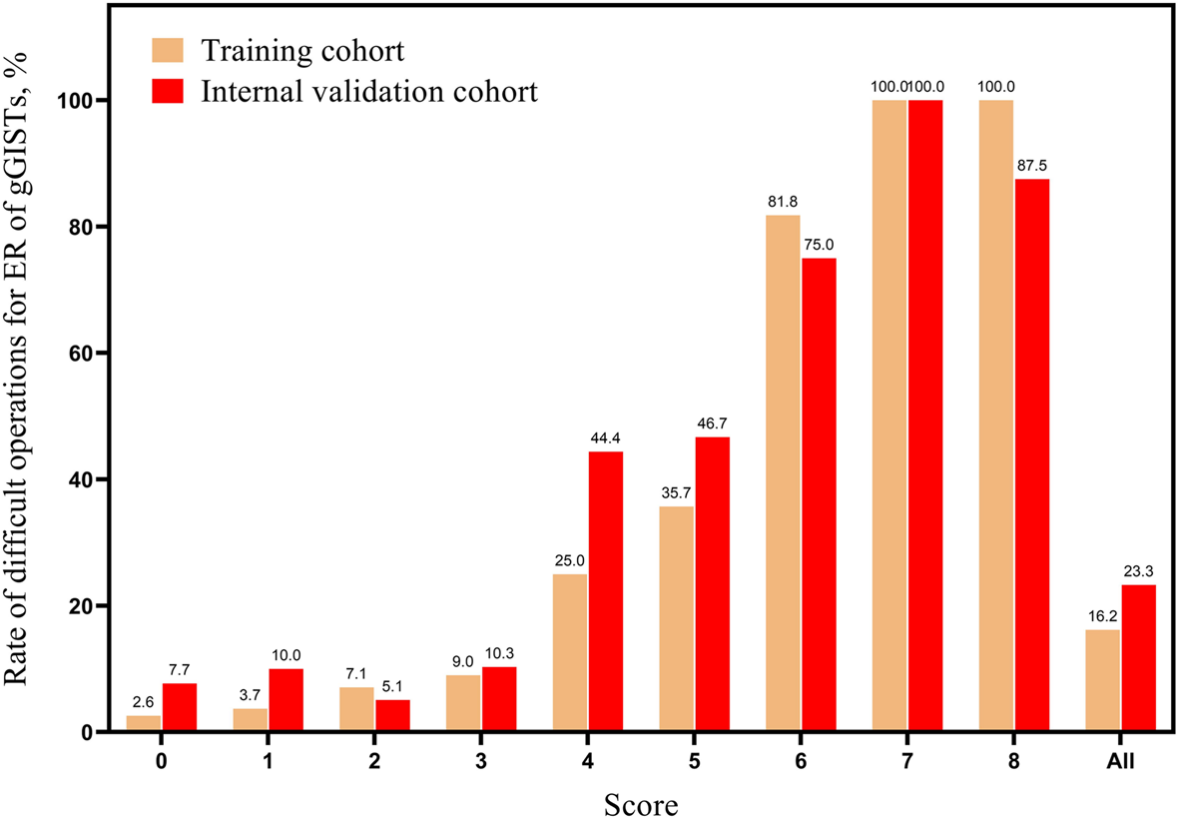
The rate of difficult operations for different scores in training cohort and internal validation cohort; gGISTs: gastric gastrointestinal stromal tumors

**Fig. 3:**
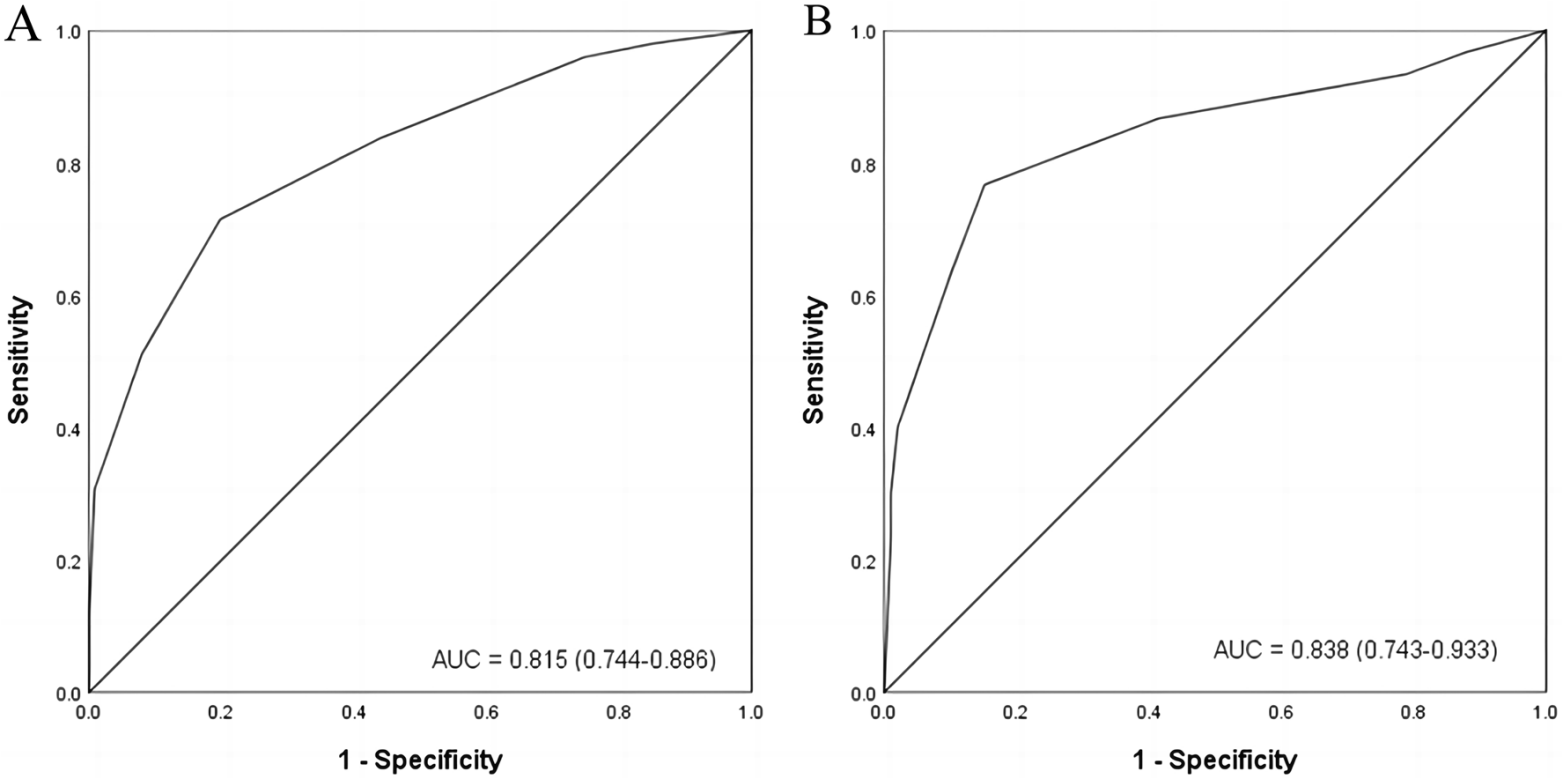
ROC curve of difficulty scoring system; **A** represent for the training cohort and **B** represent for the internal validation cohort

**Fig. 4:**
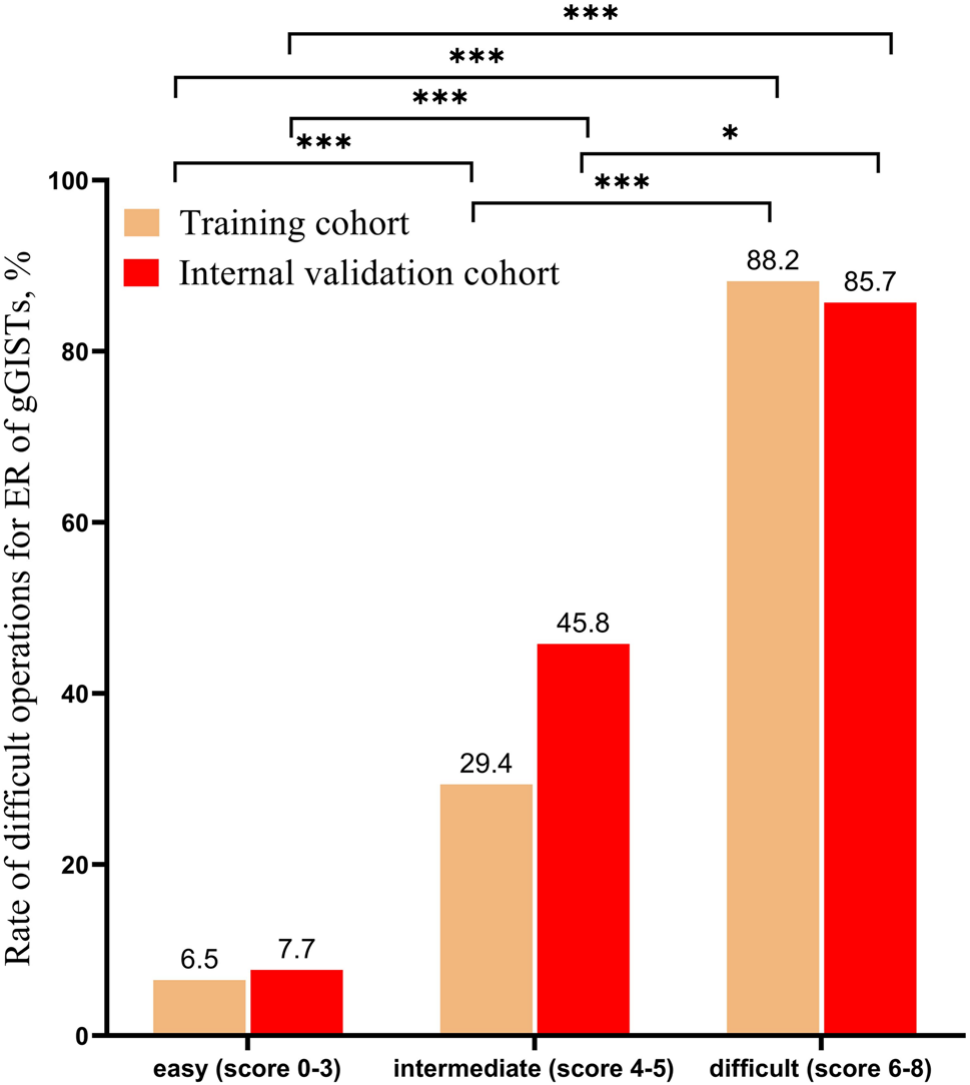
The rate of difficult operations for different grade of difficult in training cohort and internal validation cohort; gGISTs: gastric gastrointestinal stromal tumors; *P < 0.05, **P < 0.01, ***P < 0.001

We assigned a score for each risk factor based on the β-coefficient of the four independent risk factors in the multivariate analysis of the TC. The points of the four risk factors were as follows: tumor size ≥ 3.0 cm (3 points) or 2.0–3.0 cm (1 point); location in the upper third of the stomach (2 points); invasion depth beyond the muscularis propria (2 points); lack of experience (1 point) (Table 5). Figure 2 depicts the relationship between the score and the rate of difficult operations for ER of gGISTs. The rate of difficult operations for scores of 0, 1, 2, 3, 4, 5, 6, 7, and 8 in the TC were 2.6%, 3.7%, 7.1%, 9.0%, 25.0%, 35.7%, 81.8%, 100.0%, and 100.0%, respectively. The AUC for the DSS in the TC was 0.815 (95% CI 0.744–0.886, Fig. 3A), showing a good discrimination ability. The DSS was also well calibrated according to the Hosmer–Lemeshow goodness-of-fit test (c^2^ = 8.684, *P* = 0.276). The sensitivity and specificity were 0.714 and 802, and the positive predictive value (PPV) and negative predictive value (NPV) were 0.412 and 0.935. The difficulty for ER of gGISTs in the TC was then categorized into three groups: (1) easy (score 0–3); (2) intermediate (score 4–5), and (3)difficult (score 6–8). The rate of difficult operations for ER of gGISTs in each group was 6.5%, 29.4%, and 88.2%, respectively (Fig. 4).

In the IVC, the rate of difficult operations for scores of 0, 1, 2, 3, 4, 5, 6, 7, and 8 were 7.7%, 10.0%, 5.1%, 10.3%, 44.4%, 46.7%, 75.0%, 100.0%, and 87.5%, respectively (Fig. 2). The AUC for the DSS was 0.838 (95% CI 0.743–0.933, Fig. 3B), showing a good discrimination ability. The DSS was also well calibrated according to the Hosmer–Lemeshow goodness-of-fit test (c^2^ = 11.728, *P* = 0.110). The sensitivity and specificity were 0.767 and 848, and the PPV and NPV were 0.605 and 0.923. The rate of difficult operations for ER of gGISTs in 3 groups were 7.7%, 45.8%, and 85.7%, respectively (Fig. 4).

The AUC for the DSS in the EVC was 0.864 (95% CI 0.781–0.947, Supplementary Fig. 1), showing a good discrimination ability. The DSS was also well calibrated according to the Hosmer–Lemeshow goodness-of-fit test (c^2^ = 1.547, *P* = 0.992). The sensitivity and specificity were 0.889 and 651, and the PPV and NPV were 0.302 and 0.972. The rate of difficult operations for scores of 0, 1, 2, 3, 4, 5, 6, 7, and 8 were 0%, 0%, 6.5%, 17.2%, 20.0%, 42.9%, 100.0%, 66.7%, and 100.0%, respectively (Supplementary Fig. 2). The rate of difficult operations for ER of gGISTs in 3 groups were 7.0%, 29.4%, and 85.7%, respectively (Supplementary Fig. 3).

## Discussion

In the past, laparoscopic wedge gastrectomy (LWG) was the standard procedure for resecting gGISTs [21]. With the advent of ER techniques represented by ESD, ER of gGISTs has offered an opportunity to treat gGISTs at low risk [22]. Several studies have shown that ER has better short-term efficacy for gGISTs than LWG, and comparable long-term efficacy [6–8, 23, 24]. However, ER for gGISTs is more challenging, and the risk of intraoperative perforation is significantly higher than that for early gastric cancer. The probability of perforation in ER of gGISTs was about 33.3%, while the probability of perforation in ER of early gastric cancer was about 0.5–4.1% [25, 26]. Therefore, endoscopists need to master not only intraoperative hemostatic technology but also perforation repair skills to successfully complete the ER of gGISTs. In order to evaluate the difficulty for ER of gGISTs preoperative, Su et al*.* [27]constructed a Nomogram risk prediction model and showed that the tumor size, invasion depth, and the experience of the endoscopist were independent risk factors. However, a number of previous studies have shown that lesion site has an impact on the difficulty of endoscopic surgery [28–32]. Therefore, this study intends to further analyze preoperative risk factors that affect the difficulty of ER for gGIST through a multi-center and large sample size study and quantify the procedure difficulty by constructing a DSS.

Operation time, the incidence of serious intraoperative complications, and whether to transfer surgery are commonly used surrogate indicators of surgical difficulty [33–35]. Similar to the method used to define difficult procedures in Su et al*.*’s study [27] (an operative time ≥ 90 min, or the occurrence of severe intraoperative bleeding), the cases transferred to surgery were also defined as a difficult procedures in this study. After analyzing the patients, lesions, and endoscopists characteristics, we found that tumor size, location, invasion depth, and endoscopists' experience to be independent predictors that affect the difficulty for ER of gGIST. Lesions with size ≥ 2.0 cm, invasion depth beyond MP, located in the upper third of the stomach, and endoscopist's lack of experience ( ER of gGISTs < 50 cases) were more likely to undergo the difficult procedure. Based on these findings, we developed a preoperative DSS for ER of gGISTs and validated our findings in IVC and EVC. We found that the DSS we developed had good discrimination and calibration in both cohorts.

We found that tumor size is a key factor in predicting whether ER of gGISTs is difficult, with odds ratios of 2.567 and 14.271 for lesions with diameters of 2.0–3.0 cm and ≥ 3.0 cm. Previous studies have also shown that lesion size is an important factor affecting the difficulty of endoscopic surgery. Su et al*.* [27] and Jian et al*.* [28] found that ER was difficult when the tumor size was more than 3.0 cm. Due to the limited operating space of ER, the operating space and surgical field of view are worse in the treatment of gGISTs with large tumor sizes. Endoscopicists need to repeatedly adjust the angle of the endoscopic incision and the amount of air in the stomach cavity to completely remove the tumor. Moreover, larger tumors carry a higher risk of intraoperative bleeding. Endoscopists needs to have rich experience in endoscopic hemostasis to shorten the operation time and reduce the risk of transfer to laparoscopic resection. Therefore, ER of gGISTs with large tumor sizes should be performed by experienced endoscopists in order to reduce the occurrence of complications, shorten the operation time and improve the success rate of surgery.

Tumor location is also an indicator for gGISTs ER difficulty in our study. A previous study of 916 patients with early gastric cancer showed that ER required longer operative time when the lesion was located in the upper third of the stomach compared to those in the middle and lower thirds of the stomach [36]. Yoon et al*.* [31] also reported that ER of tumors in the upper third of the stomach required a longer time and had a higher risk of perforation. Wang et al*.* [37] found that ER of gGISTs located in the cardia was particularly difficult, with a higher probability of intraoperative bleeding (100%) and perforation (20%). This may be due to the special anatomical structure of the area, which often requires the endoscopist to retroflex the endoscope and consume more time, especially for doctors lacking surgical experience. Additionally, the abundance of blood vessels in the upper third of the stomach increases the risk of intraoperative bleeding. Sun et al*.* [38] compared the effectiveness of double-bend endoscopy and single-bend endoscopy in the treatment of gGISTs and found that compared with the single-bend endoscopy group, the double-bend endoscopy group had shorter operation time and a lower incidence of perforation. Due to the design of the double-curved structure, the lens can be adjusted at a larger angle so that it can be more easily close to the cardiac, the fundus of the stomach, and other special parts, so as to better expose the tumor and improve the clarity of the surgical field of vision. Therefore, for the GIST located in the upper third of the stomach, in addition to be operated by experienced endoscopists, double-curved endoscopy can be selected for conditional units.

We have found that gGISTs with invasion depth beyond the muscularis propria constitute another independent risk factor that affects the difficulty of ER, consistent with the results of Su et al*.* [27]. For lesions with deep infiltration and external growth, active perforation technique may be required to achieve complete resection, and in some cases, even intraperitoneal surgery may be necessary for tumor dissection, which greatly increases the difficulty of surgery. At the same time, when a large perforation occurs, the gastric cavity is not well filled, which seriously affects the surgical field of view under endoscopic operation, and the repair of the perforation requires more time. Therefore, for a lesion with invasion depth beyond the muscularis propria, it should be performed by experienced endoscopists. Meanwhile, direct laparoscopic resection can sometimes be considered depending on the size, location, and invasion depth of the lesion.

Surgical experience is also a crucial factor affecting the difficulty of surgery. According to Sun et al*.* [39], the learning curve of ER of gastric submucosal tumors was about 32 cases. Yoshida et al*.* [40] retrospectively analyzed the learning curve of 7 novice endoscopists in ER of gastric lesions, and the results showed that a steady state could be achieved after completing approximately 30 cases. Combining the findings of both studies, we believe that the number of cases needed to reach the learning curve may differ among endoscopists. In our study, we set a threshold of 50 patients to determine the experience level of the endoscopists in performing ER on gGISTs.

The surgical indications and specific operation steps for different endoscopic procedures vary, thereby affecting the difficulty of ER. In our study, the main endoscopic techniques used were ESD and EFTR. We found that the incidence of difficult cases in the EFTR group was higher than that in the ESD group (22.6% vs. 14.2%), and the difference between groups was statistically significant (*P* < 0.05). For tumors with external growth, EFTR is the only method for ER of gGISTs. However, for gGISTs that originate from the MP, which endoscopic procedure should be adopted and whether active perforation should be performed usually need to be judged intraoperatively. Since most gGISTs originate from the MP, endoscopic procedures cannot be defined before surgery. Therefore, in this study, endoscopic procedures were not included in the DSS. Similarly, the method of defect closure may also affect the difficulty of surgery. Smaller defects are closed with OTSC or titanium clips, while large defects require the use of nylon rope. Therefore, the closure method of the defect should be reasonably selected according to the size of the intraoperative wound surface, which could not be determined before surgery.

Our study had some limitations. First, it was a retrospective study, which may have certain selection bias and information bias. Future prospective studies with larger sample sizes can be conducted to further explore the risk factors affecting the difficulty of ER of gGISTs. Second, with the innovation of various endoscopic instruments, hemostatic materials, wound closure devices, etc., risk factors affecting the difficulty of endoscopic surgery may change, so the DSS should also keep pace with the times. Third, in this study, the risk factors of ER of gGISTs were comprehensively analyzed, but the operation steps of ESD, EFTR, and STER endoscopic techniques are different, and their respective difficulties may be different. Further research on specific endoscopic techniques may be helpful in analyzing the specific difficulties corresponding to different endoscopic techniques.

In conclusion, the DSS base on four independent risk factors (tumor size, location, invasion depth, and endoscopists' experience) was closely associated with the rate of difficult operation for ER of gGISTs. This DSS can objectively evaluate the difficulty for ER of gGISTs, which can help endoscopists select cases comparable to their own technical level and develop individualized treatment plans for cases of different difficulty.

## Supplementary Information

Below is the link to the electronic supplementary material.

Supplementary file1 Supplementary Figure 1. ROC curve of difficulty scoring system in external validation cohort. (JPG 387 KB)

Supplementary file2 Supplementary Figure 2. The rate of difficult operations for different scores in external validation cohort; gGISTs: gastric gastrointestinal stromal tumors. (JPG 483 KB)

Supplementary file3 Supplementary Figure 3. The rate of difficult operations for different grade of difficult in external validation cohort; gGISTs: gastric gastrointestinal stromal tumors; *P < 0.05, **P < 0.01, ***P < 0.001. (JPG 407 KB)

Supplementary file4 (DOCX 25 KB)
