# Supplementary figures and images for: Development and validation of a preoperative difficulty scoring system for endoscopic resection of gastric gastrointestinal stromal tumor: a multi-center study
Source: Surg Endosc. 2023 May 16;37(8):6255–66. doi: 10.1007/s00464-023-10106-w (PMC10338596; doi:10.1007/s00464-023-10106-w)

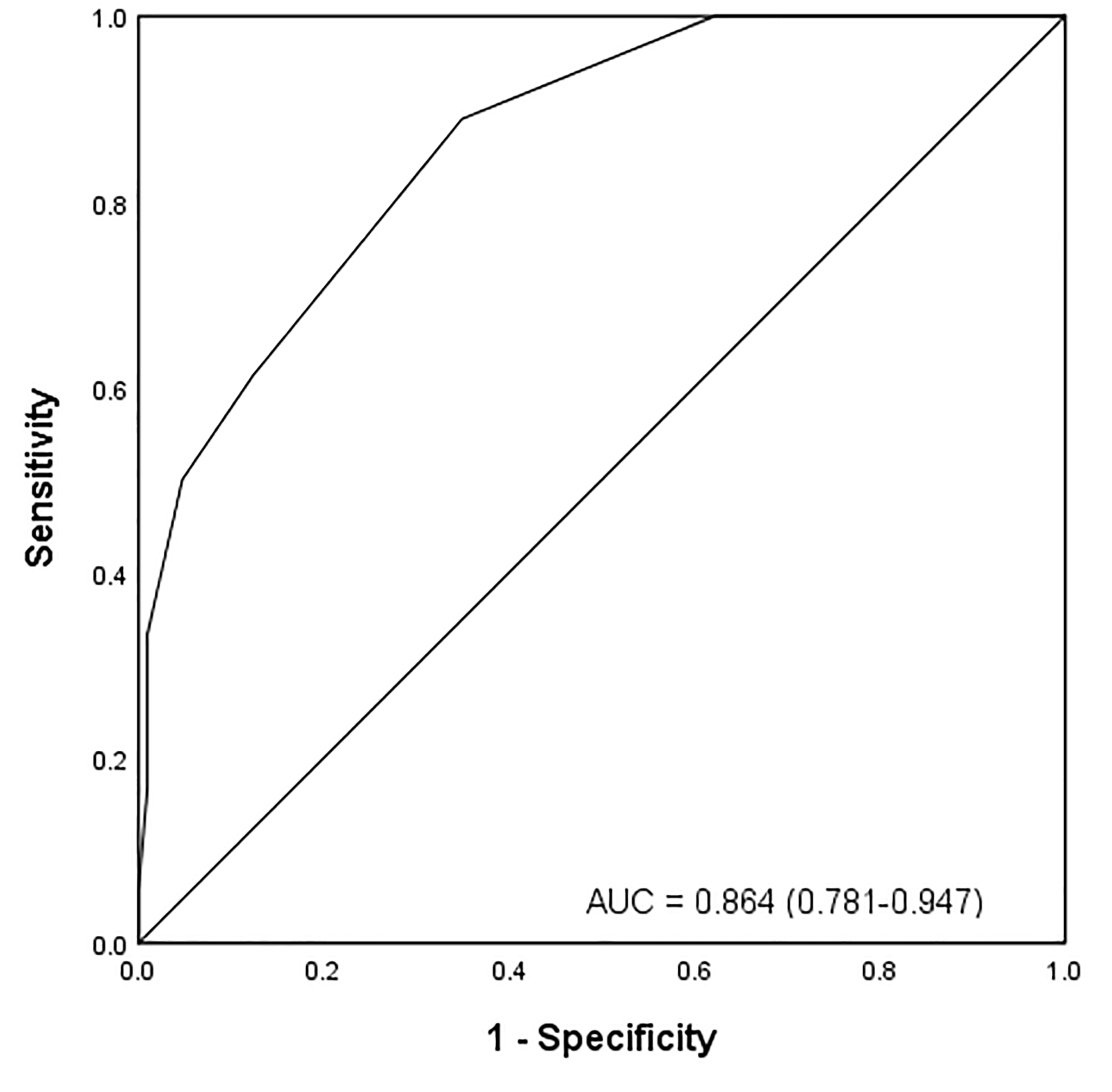

Supplement: Supplementary file 1 — Supplementary file1 Supplementary Figure 1. ROC curve of difficulty scoring system in external validation cohort. (JPG 387 KB) [file 464_2023_10106_MOESM1_ESM.jpg]

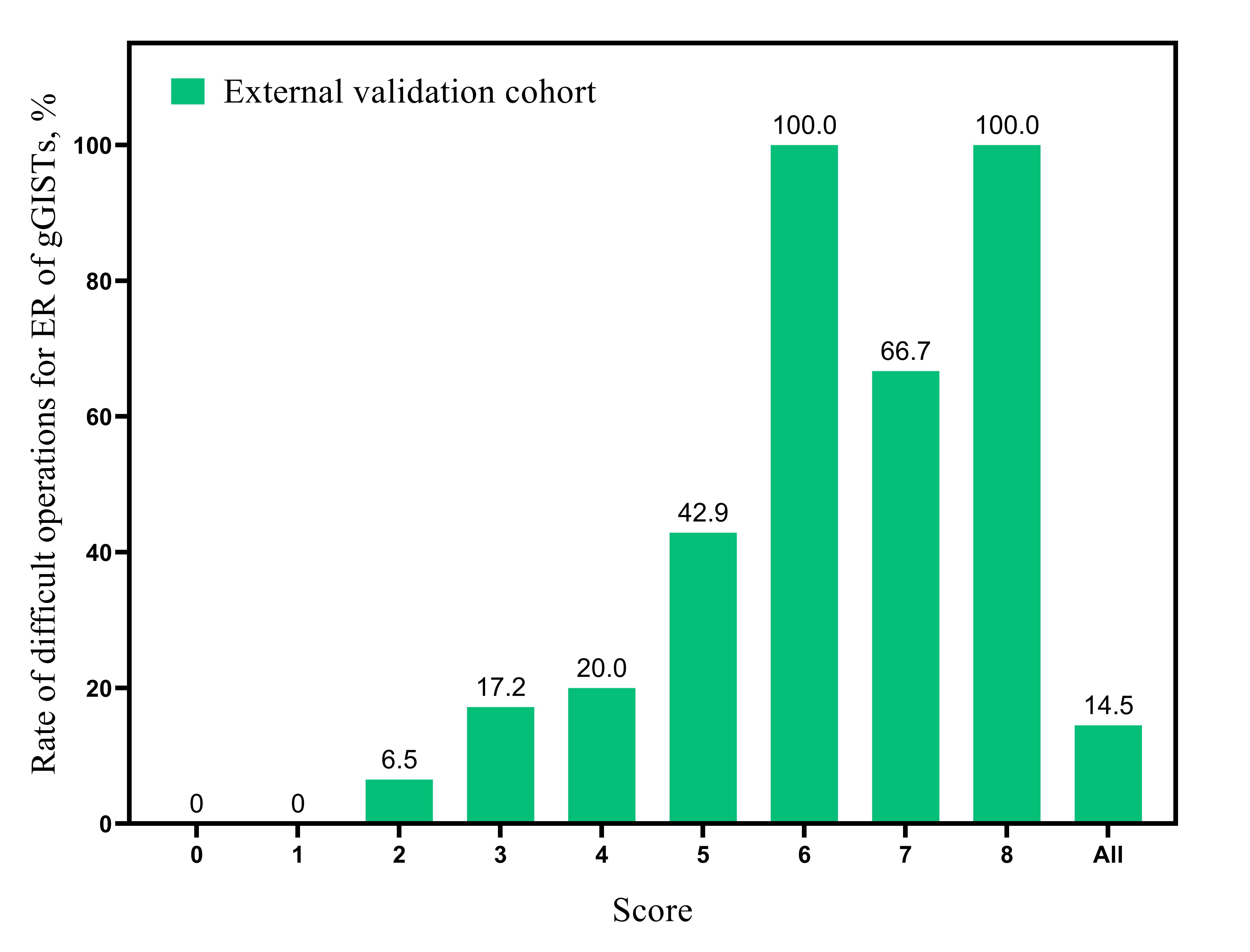

Supplement: Supplementary file 2 — Supplementary file2 Supplementary Figure 2. The rate of difficult operations for different scores in external validation cohort; gGISTs: gastric gastrointestinal stromal tumors. (JPG 483 KB) [file 464_2023_10106_MOESM2_ESM.jpg]

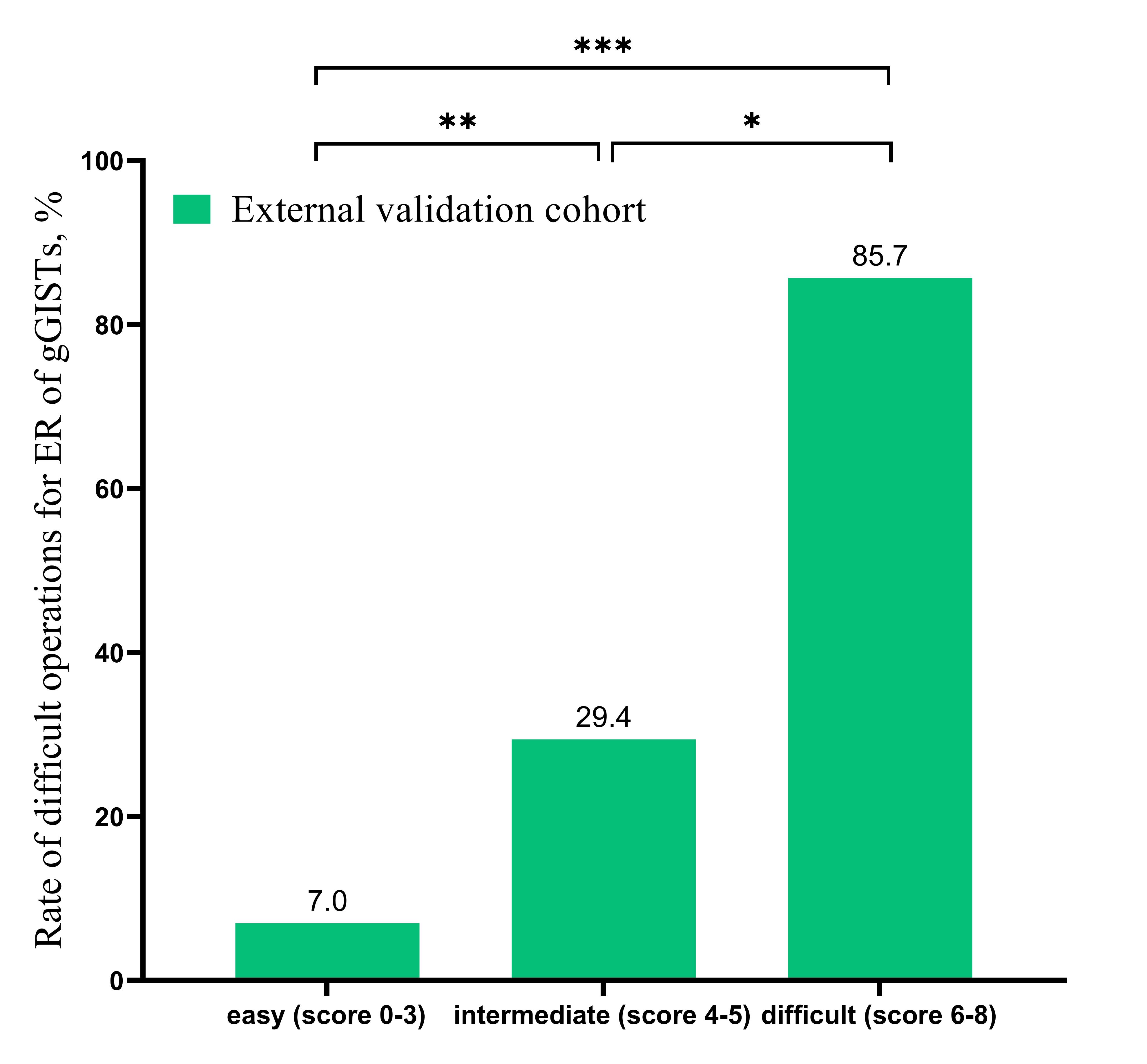

Supplement: Supplementary file 3 — Supplementary file3 Supplementary Figure 3. The rate of difficult operations for different grade of difficult in external validation cohort; gGISTs: gastric gastrointestinal stromal tumors; *P < 0.05, **P < 0.01, ***P < 0.001. (JPG 407 KB) [file 464_2023_10106_MOESM3_ESM.jpg]
