# Supplementary material for: Development and validation of a preoperative difficulty scoring system for endoscopic resection of gastric gastrointestinal stromal tumor: a multi-center study
Source: Surg Endosc. 2023 May 16;37(8):6255–66. doi: 10.1007/s00464-023-10106-w (PMC10338596; doi:10.1007/s00464-023-10106-w)
Supplement: Supplementary file 4 — Supplementary file4 (DOCX 25 KB) [file 464_2023_10106_MOESM4_ESM.docx]

**Supplementary Table 1**. Baseline characteristics of the patients and lesions between the TC and the EVC

| Variable | Training cohort  (N=302) | External validation cohort  (N=124) | *P*-value |
| --- | --- | --- | --- |
| Gender, n (%)  Male  Female | 145（48.0）  157（52.0） | 46（37.1）  78（62.9） | 0.040 |
| Age, yesrs, n (%)  < 60  ≥ 60 | 147（48.7）  155（51.3） | 60（48.4）  64（51.6） | 0.957 |
| Primary symptom, n (%)  Asymptomatic  Abdominal discomfort  Hemorrhage | 65（21.5）  230（76.2）  7（2.3） | 30（24.2）  92（74.2）  2（1.6） | 0.743 |
| Smoking, n (%)  Yes  No | 92（30.5）  210（69.5） | 38（30.6）  86（69.4） | 0.971 |
| History of drinking, n (%)  Yes  No | 67（22.2）  235（77.8） | 28（22.6）  96（77.4） | 0.929 |
| Hypertension, n (%)  Yes  No | 98（32.5）  204（67.5） | 40（32.3）  84（67.7） | 0.969 |
| Coronary disease, n (%)  Yes  No | 55（18.2）  247（81.8） | 23（18.5）  101（81.5） | 0.935 |
| Diabetes, n (%)  Yes  No | 75（24.8）  227（75.2） | 31（25.0）  93（75.0） | 0.971 |
| ASA score, n (%)  I  II  III | 253（83.8）  49（16.2）  0 | 106（85.5）  18（14.5）  0 | 0.666 |
| BMI, kg/m², n (%)  < 18.5  18.5-23.9  ≥ 24.0 | 53（17.5）  144（47.7）  105（34.8） | 26（21.0）  62（50.0）  36（29.0） | 0.466 |
| Location, n (%)  Upper  Middle  Lower | 210（69.5）  61（20.2）  31（10.3） | 74（59.7）  40（32.3）  10（8.1） | 0.028 |
| Location, n (%)  Lesser curvature  Greater curvature  Anterior  Posterior | 107（35.4）  25（8.3）  116（38.4）  54（17.9） | 32（25.8）  13（10.5）  51（41.1）  28（22.6） | 0.246 |
| Shape, n (%)  Regular  Irregular | 273（90.4）  29（9.6） | 107（86.3）  17（13.7） | 0.215 |
| Invasion depth, n (%)  MM  MP  MP-ex | 70（23.2）  190（62.9）  42（13.9） | 17（13.7）  85（68.5）  22（17.7） | 0.076 |
| Boundary, n (%)  Clear  Unclear | 269（89.1）  33（10.9） | 92（74.2）  32（25.8） | <0.001 |
| Size, cm, n (%)  ≥ 3.0  2.0-3.0  < 2.0 | 42（13.9）  85（28.1）  175（57.9） | 10（8.1）  24（19.4）  90（72.6） | 0.017 |

TC: training cohort; EVC: external validation cohort; ASA: American Society of Anesthesiologists; BMI: body mass index; MM: muscularis mucosae; MP: muscularis propria; MP-ex:MP with exophytic growth

**Supplementary Table 2**. Distribution of cases defined as difficult procedure in external validation cohort

|  | External validation cohort  (N=124) |
| --- | --- |
| Difficult procedure, n (%)  Long operative time, n (%)  Severe intraoperative bleeding, n (%)  Conversion, n (%) | 18 (14.5)  16 (12.9)  4 (3.2)  0 |

**Supplementary Table 3**. Procedural outcomes related to ER of gGISTs between the TC and the EVC

| Variable | Training cohort  (N=302) | External validation cohort  (N=124) | *P*-value |
| --- | --- | --- | --- |
| Experience, cases, n (%)  < 50  ≥ 50 | 94（31.1）  208（68.9） | 46（37.1）  78（62.9） | 0.233 |
| Endoscopic tecnique, n (%)  ESD  EFTR  STER | 159（52.6）  138（45.7）  5（1.7） | 62（50.0）  60（48.4）  2（1.6） | 0.893 |
| Modified NIH risk criteria, n (%)  Very low  Low  Intermediate  High | 205（67.9）  65（21.5）  27（8.9）  5（1.7） | 86（69.4）  24（19.4）  12（9.7）  2（1.6） | 0.967 |
| Operative time, min, median(IQR) | 59.0（45.0，74.3） | 68.0（50.0，75.0） | 0.016 |
| Conversion, n (%) | 10（3.3） | 0 | 0.039* |
| Severe intraoperative bleeding, n (%) | 8（2.6） | 4（3.2） | 0.744 |
| Postoperative hospitalization, days, median(IQR) | 6.0（5.0，7.0） | 5.0（5.0，7.0） | 0.051 |
| Postoperative fasting, days, median(IQR) | 3.0（2.0，3.0） | 2.0（2.0，3.0） | 0.086 |
| R0 resection, n (%) | 275（91.1） | 108（87.1） | 0.217 |
| Postoperative complications, n (%) | 46（15.2） | 16（12.9） | 0.536 |

TC: training cohort; EVC: external validation cohort; ER: endoscopic resection; gGIST: gastric gastrointestinal stromal tumor; ESD: endoscopic submucosal dissection; EFTR: endoscopic full-thickness resection; STER: submucosal tunnel endoscopic resection; NIH: National Institute of Health; *Fisher’s exact test
